# Ophthalmic complications of endoscopic ethmoidectomy: a case report

**DOI:** 10.1186/1757-1626-3-63

**Published:** 2010-02-18

**Authors:** Iwona Niedzielska, Tadeusz Cieslik, Tomasz Janic

**Affiliations:** 1 Department of Craniomaxillofacial Surgery, Silesian Medical University, ul. Francuska 20/24, 40-027 Katowice, Poland

## Abstract

Damage to ethmoid structures following removal of nasal polyps can cause severe complications. A patient aged 48 years with damage to right orbital structures sustained in the course of transnasal endoscopic surgery for ethmoid polyps was operated. After operation were complications.

## Background

Nasal polyposis is a result of chronic inflammation of the nasal mucous membrane. The etiology remains unknown and is still studied. Nasal polyps are present in around 4% of the population. Their formation is observed in chronic sinusitis, environmental allergies, and aspirin idiosyncrasy causing nasal obstruction, permanent rhinorrhea, headaches and facial pain, which all reduce quality of life. The management of nasal polyps may involve medical or surgical therapy, which should be based on meticulous diagnostic work-up of the patients. The recurrence rate of nasal polyps is can be even higher than 30% [1]. Complications have been observed following removal of nasal polyps located in the area of ethmoid structures; these are separated from the orbit by extremely thin lamina papyracea (or orbital lamina), and from the anterior cranial fossa by the cribriform plate, which is also thin. Damage to the structures can cause severe complications occurring in 1% of surgical patients [2].

## Case presentation

**Figure 1:**
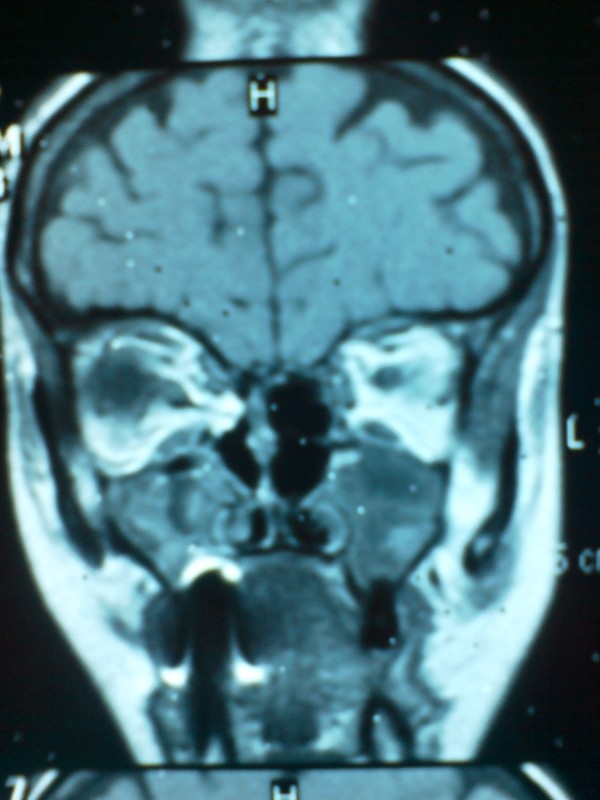
**CT scan**. Defect in the medial orbital wall.

**Figure 2:**
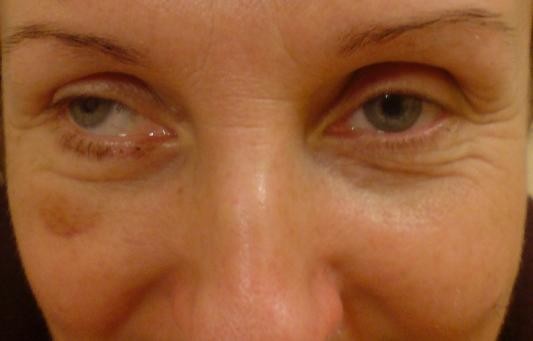
**The result of surgery**.

A patient aged 48-years (patient's initials LE, medical records 08/65c, white female) was admitted to the Department of Craniomaxillofacial Surgery in Katowice in January 2008 with damage to right orbital structures sustained in the course of transnasal endoscopic surgery for ethmoid polyps. The procedure had been performed in a national health service centre two weeks before, due to problems with nasal breathing, and persistent discharge. Discharge information included iatrogenic orbital haemorrhage and damage to right medial rectus muscle. Following recovery from anaesthesia, the patient complained of vision loss of several hours duration. Emergency repeat surgery was performed to control bleeding, but the medial orbital wall defect and damage muscle were not repaired. The patient underwent NMR and CT imaging, was referred to an ophthalmic ward and treated for two weeks with Dexamethason and Mannitol, the medical documentation about the eye fundus investigation are unavailable. Imaging investigations revealed a 12 × 8 mm defect in the medial orbital wall and prolapse of adipose tissue into the nasal cavities. Medial rectus muscle mechanical injury and thinning were also observed (Figure. 1). On admission to the Department of Craniomaxillofacial Surgery the patient showed restriction of ocular motility, diplopia, and eyelid bruising, there were no visual acuity disturbances. The patient was operated on under general anaesthesia; a 1 × 15 mm medial orbital wall defect was confirmed and repaired using iliac crest graft. Diplopia subsided. Despite ocular movements remaining unsynchronised due to medial rectus injury, the patient is content with the result of surgery (Figure. 2). Orthoptic rehabilitation caused slight improvement. The patient is under regular ophthalmic checkups; botulinus toxin administration to medial rectus antagonist is considered.

## Conclusion

**Figure 3:**
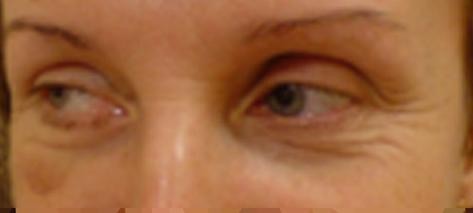
**The head movement responses are performed to compensate for vision disturbances**.

In patients who fail medical therapy nasal polyposis can be effectively treated using ethmoidectomy or endoscopic surgery [3]. However, at the beginning of the century, ethmoidectomy was considered one of those surgery types that may cause severe trauma to the patient. Serious complications include intracranial haematoma, massive haemorrhage, orbital haematoma, blindness, damage to the dura mater [4] with resulting death of the patient. Rare complications are orbital oedema and worsening of olfactory function. Recently, and strictly speaking for 20 years now, the use of modern equipment including endoscopes, has allowed a significant decrease in complication rates after nasal polypectomies. Stankiewicz [2] gave only 6% and 13% rates of severe and slight complications, respectively. Desii et al [5], noted a complication rate as low as 1.2%. Freedman and Keer [6], performed 1000 ethmoidectomies in 565 patients; they observed mild complications in 26 patients only, and no cases of death or blindness. The authors emphasize the fact that new surgical techniques minimize the risk of complications following operations that in the past could cause injury or even death of the patient. Dalziel et al. [7] attempted a review of literature on nasal polypectomy. They found significant discrepancies between authors' opinions; a wide range of injuries and complications, also severe, shows that endoscopic surgery cannot be considered a safe means of polyp removal. Our case confirms the conclusion although endoscopic surgery rarely yields orbital complications. Dunya et al. [8] had 5 patients with such complications following a total of 372 ethmoidectomies. Ocular complications including blindness or diplopia can result from iatrogenic injuries affecting the nerves or muscles of the eyeball or from retrobulbar haematoma. Damage to anterior or posterior ethmoid vessels causes bleeding and a resulting enophthalmos. Only timely surgical intervention, a 24-hour monitoring of the patient's condition, and ophthalmic follow-up can prevent further complications. Intervention performed in our patient prior to admission to the Department of Craniomaxillofacial surgery caused intraorbital bleeding and vision loss. This was followed by diplopia and eyeball motility limitations resulting from damage to the right medial rectus. Medial orbital wall repair did not bring a fully satisfactory result as the eyes remain nonsynchronized in their movements. The patient's head movement responses are performed to compensate for vision disturbances (Figure. 3). Botulinus toxin administration to medial rectus antagonist is considered as in the report of Hong et al. [9].

## Consent

Written informed consent was obtained from the patient for publication of this case report and accompanying images. A copy of the written consent is available for review by the Editor-in-Chief of this journal.

## Competing interests

The authors declare that they have no competing interests.

## Authors' contributions

NI did examination of the patient, manuscript edition, references, CT and JT involved in manuscript edition.

All authors read and approved the final manuscript.

## References

[B1] Chandra RK Brol BM Immunopathology of nasal polypi J Laryngol Oto 1974 88 1019 1024 10.1017/s0022215100079676 4470574

[B2] Stankiewicz JA Complications of endoscopic nasal surgery: occurrence and treatment Am J Rhinol 1987 1 45 49 10.2500/105065887781390417

[B3] Jurkiewicz D Present opinions on treatment of nasal polyps Pol Merkuriusz Lek 2006 20 119 591 597 16875170

[B4] Weber R Draf W Complications of endonasal micro-endoscopic ethmoid bone operation HNO 1992 40 5 170 175 1612932

[B5] Dessi P Casto F Triglia JM Zanaret M Cannoni M Major complications of sinus surgery: a review of 1192 procedures J Laryngol Otol 1994 108 212 215 8169501 10.1017/s0022215100126325

[B6] Freedman HM Kern EB Complications of intranasal ethmoidectomy: a review of 1,000 consecutive operations Laryngoscope 1979 89 3 421 434 10.1288/00005537-197903000-00010 431247

[B7] Dalziel K Stein K Round A Garside R Royle P Endoscopic sinus surgery for the excision of nasal polyps: A systematic review of safety and effectiveness Am J Rhinol 2006 20 5 506 519 10.2500/ajr.2006.20.2923 17063747

[B8] Dunya IM Salman SD Shore JW Ophthalmic complications of endoscopic ethmoid surgery and their management Am J Otolaryngol 1996 17 5 322 331 10.1016/S0196-0709(96)90019-8 8870938

[B9] Hong JE Goldberg AN Cockerham KP Botulinum toxin A therapy for medial rectus injury during endoscopic sinus surgery Am J Rhinol 2008 22 1 95 97 10.2500/ajr.2008.22.3123 18284867

